# Using a Virtual Curriculum to Provide Interprofessional Student Education in Alzheimer’s Disease

**DOI:** 10.1093/geroni/igaa057.013

**Published:** 2020-12-16

**Authors:** Alyssa Indelicato, Lauren Germain, Liliana Barros Una, Alice Krueger, Ann Ludwig, Sharon Brangman, Telisa Stewart

**Affiliations:** 1 SUNY Upstate Medical University, Syracuse, New York, United States; 2 SUNY Upstate Medical University, Syracuse, United States; 3 Virtual Ability, Inc., Aurora, Colorado, United States

## Abstract

As Alzheimer’s disease increases in prevalence among older adults, there is an increased need for health professionals to effectively communicate with patients and their caregivers. Interactions between providers and Alzheimer’s patients differ from a typical patient-physician encounter. The project created a virtual learning environment (VLE) to better prepare students to engage with Alzheimer's patients and their caregivers. The VLE includes a clinical space, clinical office, and a patient’s home environment for students to practice their health communication skills and to better understand the lived experience. Nursing, MD, and Physician Assistants students were recruited and assigned a virtual provider (avatar). Students engage with a virtual patient (bot) in an exam room and then are directed to engage with a virtual caregiver (bot) in a clinical office. The virtual patient and caregiver have been programmed to respond to questions respectively and provide detailed insight into their lived experiences. After these encounters, the student is asked to react on the information obtained. The student is asked to explore the virtual home environment and triangulate the home experience, the caregiver’s perspective, and the patient’s responses. We are enrolling students to participate in the project and will complete the evaluation data. Students will be assessed for knowledge, self-efficacy, and their overall satisfaction with the virtual learning environment. We anticipate students will have an increase in each of these areas and will have improved communication skills with this at-risk, older adult population.

